# Assessment of the Enrichment of Heavy Metals in Coal and Its Combustion Residues

**DOI:** 10.1021/acsomega.2c02308

**Published:** 2022-06-09

**Authors:** Aydan Altıkulaç, Şeref Turhan, Aslı Kurnaz, Elif Gören, Celalettin Duran, Aybaba Hançerlioğulları, Fatma Aysun Uğur

**Affiliations:** † Ula Ali Koçman Vocational School, Muğla Sıtkı Koçman University, 48640 Ula, Muğla, Turkey; ‡ Department of Physics, Faculty of Science and Letters, Kastomunu University, 37150 Kastamonu, Turkey; § Department of Physics, Faculty of Science and Letters, Korkut Ata University, 80010 Osmaniye, Turkey; ∥ Department of Geography, Science and Letters Faculty, Kastamonu University, 37150 Kastamonu, Turkey

## Abstract

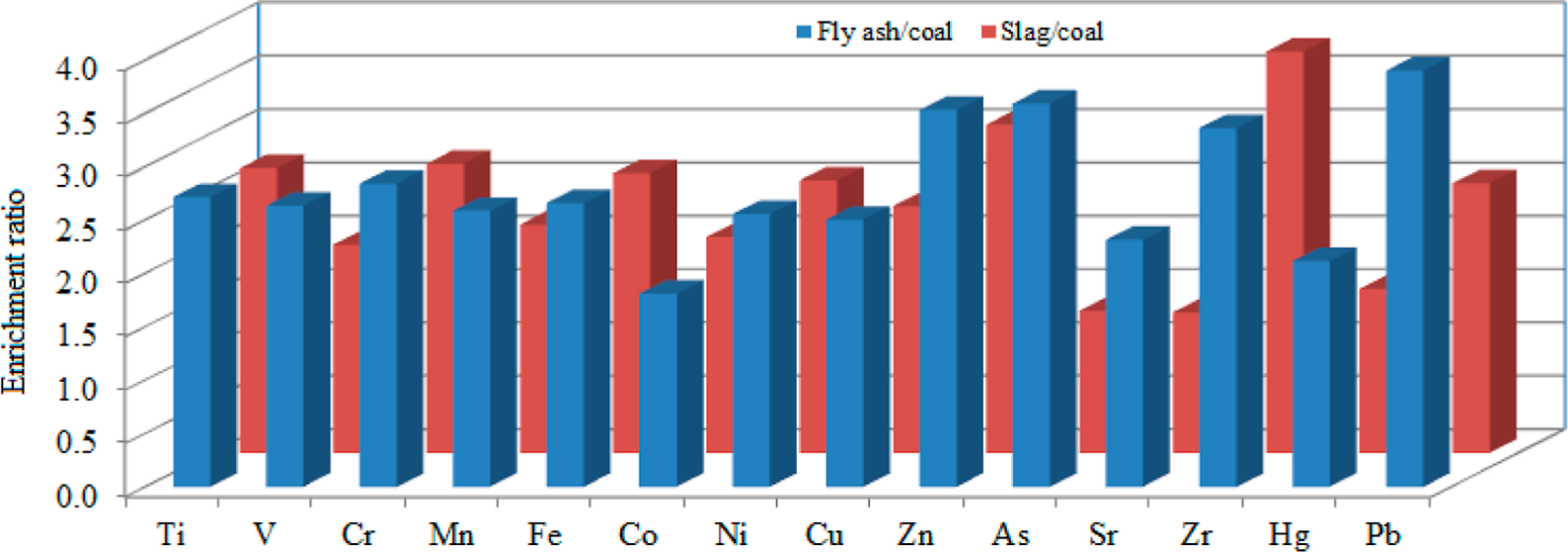

Coal-fired thermal power plants remain one of the main sources of electricity generation in Turkey. Combustion of coal creates coal ash and slag, which are often stored in landfills located near residential and agricultural fields, increasing the potential for high environmental contamination and health risks. This study investigates the content and enrichment factor (EF) of heavy metals in pulverized lignite coal and its combustion residues from the Kangal lignite coal-fired thermal power plant situated in the Central Anatolian Region of Turkey. The concentration of heavy metals (Ti, V, Cr, Mn, Fe, Co, Ni, Cu, Zn, As, Sr, Zr, Cd, Hg, and Pb) in lignite coal, slag, and fly ash samples were analyzed using an energy dispersive X-ray fluorescence technique. The concentration of Fe is highest while Hg concentration is lowest in the samples. The concentrations of heavy metals are higher in slag and fly ash samples than in lignite coal. Average values of EF (related to Earth’s crust average) revealed that extreme enrichment has been shown by arsenic and mercury in lignite coal and fly ash samples while very high enrichment has been shown in slag samples.

## 1 Introduction

Thermal power plants (TPPs) generate electricity using fossil fuels such as coal, gas, and oil. In Turkey, coal is the third-largest primary energy source, representing 28% of the total primary energy supply in 2019 because Turkey has large domestic coal resources.^1^ Domestic coal, mainly lignite production, covers 71% of the total coal supply in terms of mass.^1^ In Turkey, the production of lignite with a low calorific value compared to hard coal and imported steam coal reserves is estimated to be approximately 17.5 billion tons, which corresponds to approximately 2.1% of the total world coal reserves.^2^ Therefore, the number of coal-fired power plants (CFTPPs) has grown rapidly in recent years, accounting for more than a third of electricity production in 2019 and contributing to about half of the total growth in electricity generation over the past decade.^1^ As of May 2020, the total installed power capacity of CFTPPs was 20.3 GW (10,097 MW lignite, 810.8 MW hard coal, 405 MW asphaltite, and 8, 967 MW imported coal), which is equal to 22% of the total installed power capacity of Turkey.^1^ As part of its strategy to reduce dependency on imported energy sources, Turkey plans to install new lignite-fired TPPs (LFTPPs) with a total power of 7.5 GW by 2027.^1^

Large-scale utilization of pulverized coal in industrial power generation produces not only acidic pollutants (SO_2_ and NO_x_) but also significant amounts of combustion residues (wastes) or by-products such as pulverized coal ash and slag.^3,4^ Currently, millions of tons of pulverized coal ash (bottom ash of approximately 20% and fly ash of approximately 80%) and slag are produced from more than 30 CFTPPs installed in Turkey. Depending on the efficiency of the electrostatic precipitators, most of the fly ash (approximately 99.5%) is collected, while the remainder is released into the atmosphere.^5^ Most of these pulverized coal ashes are disposed of on the land or in the ash ponds.^6^ Only a very small fraction of these coal ashes is used, and the utilization rate of fly ash (1%) is significantly below the global utilization rate (25%).^6,7^ During pulverized coal combustion, heavy metals or potentially toxic elements (As, Cr, Zr, Ni, Cd, Hg, Pb, etc.) are redistributed into electrostatic precipitator fly ash, bottom ash, and slag. Fly ash captures most of the heavy metals, so it is considered the most important combustion residue.^8^ The accumulation and concentration of these heavy metals in fly ash depend on the feed coal, combustion methods, and pollution control equipment at a facility.^8,9^

Some heavy metals that can be transported or leached out by atmospheric mobilization from fly ash, bottom ash, and slag stored in large fillings can contaminate soil, surface, and groundwater. As a result, from an environmental and human health point of view, CFTPP is considered a major source of heavy metals in the environment and represents serious environmental hazards. Therefore, the determination of concentration and speciation of trace heavy metals released from coal combustion is very important for the assessment of health and environmental risks.^3,10^ To date, many research studies have been conducted on the determination of concentration, speciation, and characterization of heavy metals in coal and combustion residues of CFTPPs.^3,7,10−25^ However, a limited number of studies have been performed on the heavy metals found in coal and its wastes used in CFTPPs installed in Turkey. Ertuğrul et al.^26^ determined the concentrations of As, Sr, Mo, Ba, In, and Ce in fly ash from Afsin-Elbistan LFTPP. Baba^27^ analyzed the concentrations of 23 trace (Ag, As, Ba, Be, Bi, Cd, Co, Cu, Cr, La, Mn, Mo, Ni, Pb, Sb, Sn, Sr, Ti, V, W, Y, Zr, and Zn) and 7 major (, Al, Ca, Na, K, Mg, Fe, and P) elements in coal and its wastes (fly and bottom ash) from Yatağan LFTPP in the western part of the Aegean region. Dogan et al.^28,29^ determined the concentrations of Nd, Ba, Sr, Mo, and As in fly ash samples from Yeniköy LFTPP and Kemerköy LFTPP. Dogan and Kobya^30^ determined the concentrations of Sn, La, Ba, Sr, Zr, and Mo in fly ash samples from Yatağan LFTPP. Sutcu and Karayiğit^31^ investigated major and trace element concentrations of coal samples from Afsin-Elbistan LFTPP. Karayiğit et al.^32^ analyzed major and trace elements (Al, Ca, Fe, K, Mg, Mn, Na, Ti, S, As, B, Ba, Be, Bi, Cd, Co, Cr, Cu, Cs, Ga, Ge, Hf, Hg, Li, Mo, Nb, Ni, P, Pb, Rb, Sb, Sc, Se, Sn, Sr, Ta, Th, Tl, U, V, Y, Zn, Zr, and REEs) in coal and its residues (fly and bottom ash) from Soma LFTPP. According to our literature search, there is no detailed study on the determination of the concentrations of heavy metals in coal and its residues from Kangal LFTPP with a power of 457 MW, which corresponds to approximately 5% of the total installed power of LFTPPs in Turkey. Because the thermal quality of the lignite coal used in Kangal LFTPP is very low and the ash content is very high, it is important for human and environmental health to analyze the potentially toxic elements of millions of solid wastes generated every year. The objectives of this study are to (i) analyze the concentration of heavy metals and metalloids (Ti, V, Cr, Mn, Fe, Co, Ni, Cu, Zn, As, Sr, Zr, Cd, Hg, and Pb) in lignite coal (LC), lignite slag (LS), and fly ash (FA) samples from Kangal LFTPP using an energy dispersive X-ray fluorescence (EDXRF) technique and (ii) estimate enrichment ratio (ER) (to coal average) and enrichment factor (EF) (to Earth’s crustal average) of the heavy metals. This study represents the first attempt to determine the heavy metal contents of the lignite coal used in Kangal LFTPP and LC, LS, and FA samples obtained as wastes from the power plant and to estimate the ERs and EFs of these metals.

## 2 Materials and Methods

### 2.1 Site

**Figure 1:**
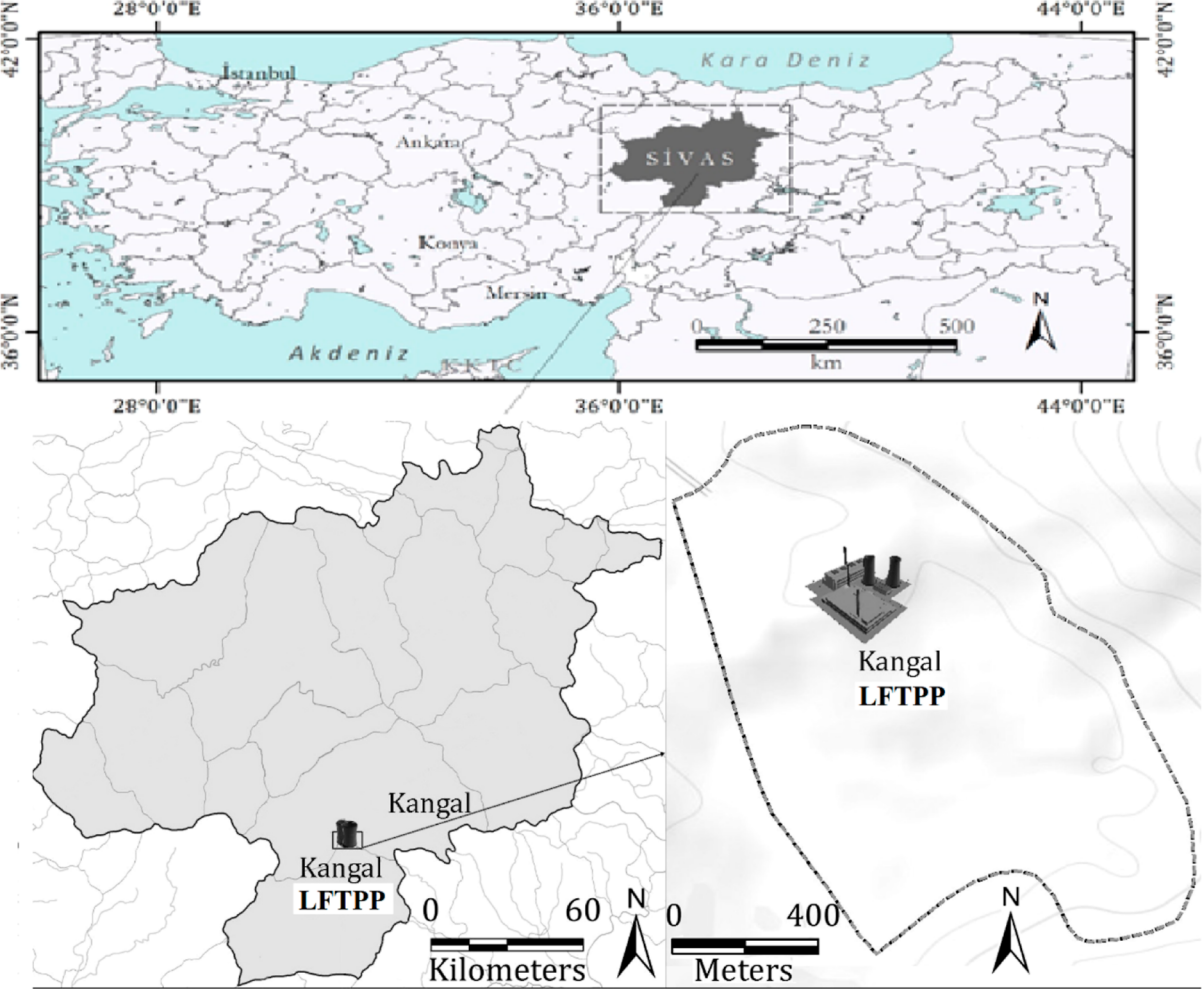
Map of Kangal lignite-firing TPP.

Kangal LFTPP has a 457 MW (2 × 150 MW + 1 × 157 MW) capacity and has been working since 1989. It is situated in the Hamal Village of Kangal County, in the south of Sivas province, located in the Central Anatolia Region of Turkey (Figure 1). The population of the county is 20,760, and it has an extension of 3224 km^2^. There are lignite coal deposits about 25 km south of the county. Kangal LC has an average calorific value of 1100 kcal kg^–1^, an average sulfur content of 3%, an average moisture content of 51%, and a fly ash fraction of 21%.^33^ The annual lignite consumption of Kangal LFTPP is approximately 7 million tons. Two units of the TPP are equipped with electrostatic precipitators for FA collection. In 2019, approximately 1.8 million tons of FA and 1.7 thousand tons of LS were obtained as residues or by-products.^34^ The fly ashes kept in electrostatic filters and the slag falling under the boiler and cooled in a water-filled vat are collected in separate silos and then transported to an ash mountain in a valley located 1200 m southwest of the power plant by belts in the closed gallery.^34^

### 2.2 Sampling, Sample Preparation, and Heavy Metal Analysis

Ten samples of LC, LS, and FA were collected from different parts of the lignite basin, the slag storage field, and the ash mountain of Kangal LFTPP, respectively. Kangal LC has high humidity (43.1–55.8%) and volatile matter content (37.7–46.2%).^35^ Kangal LS generally has a uniform grain size (0.5–0.5 mm) and a smooth surface texture. Kangal FA is classified as calcareous FA because its reactive lime is over 10%. Kangal FA consists of a mixture of glassy and crystalline phases. In the morphological examination of Kangal FA, it was observed that it contained agglomerated particles ranging in size from 1 to 100 μm.^36^ LC and LS samples were grounded and powdered to make them fit the calibrated powder geometry.^2^ Then, each sample was dried and homogenized, and approximately 5 g of each sample was taken for analysis.

EDXRF spectrometric method is known as a fast, reliable, accurate, precise, and repeatability analysis method. It is used for qualitative and quantitative multi-element analysis of major, minor, and trace element concentrations in environmental samples and requires minimal sample preparation.^19,37,38^ The collected samples were analyzed for the following heavy metals: Ti, V, Cr, Mn, Fe, Co, Ni, Cu, Zn, As, Sr, Zr, Hg, and Pb. Analysis of concentrations of the heavy metals was carried out using an EDXRF spectrometer (SPECTRO XEPOS) equipped with a thick binary Pd/Co alloy anode X-ray tube (50 kV, 60 W).^2^ Detailed information about the EDXRF spectrometer was given in detail in the study by Turhan et al.^2^ The EDXRF spectrometer optimizes the excitation using polarization and secondary targets.^2^ The spectrometer has an autosampler for up to 12 items and software modules. The target modifier with up to eight polarizations and secondary targets offers many different excitation conditions, ensuring optimal detection of all elements from K to U.^2^ The EDXRF spectrometer uses advanced calibration techniques such as “non-standard” calibration, often based on the fundamental parameter method. TurboQuant II software quickly and accurately analyzes practically any unknown liquid, powder, or solid sample. Soil-certified reference material (NIST SRM 2709) was used for the quality assurance of the EDXRF system.^2^ The sample containers prepared for each sample were placed in the automatic sampler and counted once in 2 h, and the analysis was completed. The detection limits of Ti, V, Cr, Mn, Fe, Co, Ni, Cu, Zn, As, Sr, Zr, Hg, and Pb were determined as 2.0, 3.0, 1.0, 1.0, 1.0, 3.0, 0.5, 0.5, 0.5, 0.5, 3.2, 1.0, 1.0, and 1.2 mg/kg, respectively.

## 2.3 Enrichment Factor

The EF to Earth’s crustal average was used to evaluate the degree of heavy metal pollution and estimated using the following formula^2,19^
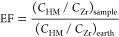
1where *C*_HM_ and *C*_Zr_ are the concentration of heavy metals and Zr in the sample and Earth’s crust, respectively. In the calculation of EF, Zr is taken as a reference element that is supposed to be practically not released by human activities.^39^ The five contamination categories are recognized on the basis of the value of EF:^40^ EF < 2, depletion to slightly enrichment; 2 ≤ EF < 5, moderate enrichment; 5 ≤ EF < 20, significant enrichment; 20 ≤ EF < 40, very high enrichment; and EF ≥ 40, extremely enrichment.

## 3 Results and Discussion

### 3.1 Major and Minor Oxides in Slag and Fly Ash

**Figure 2:**
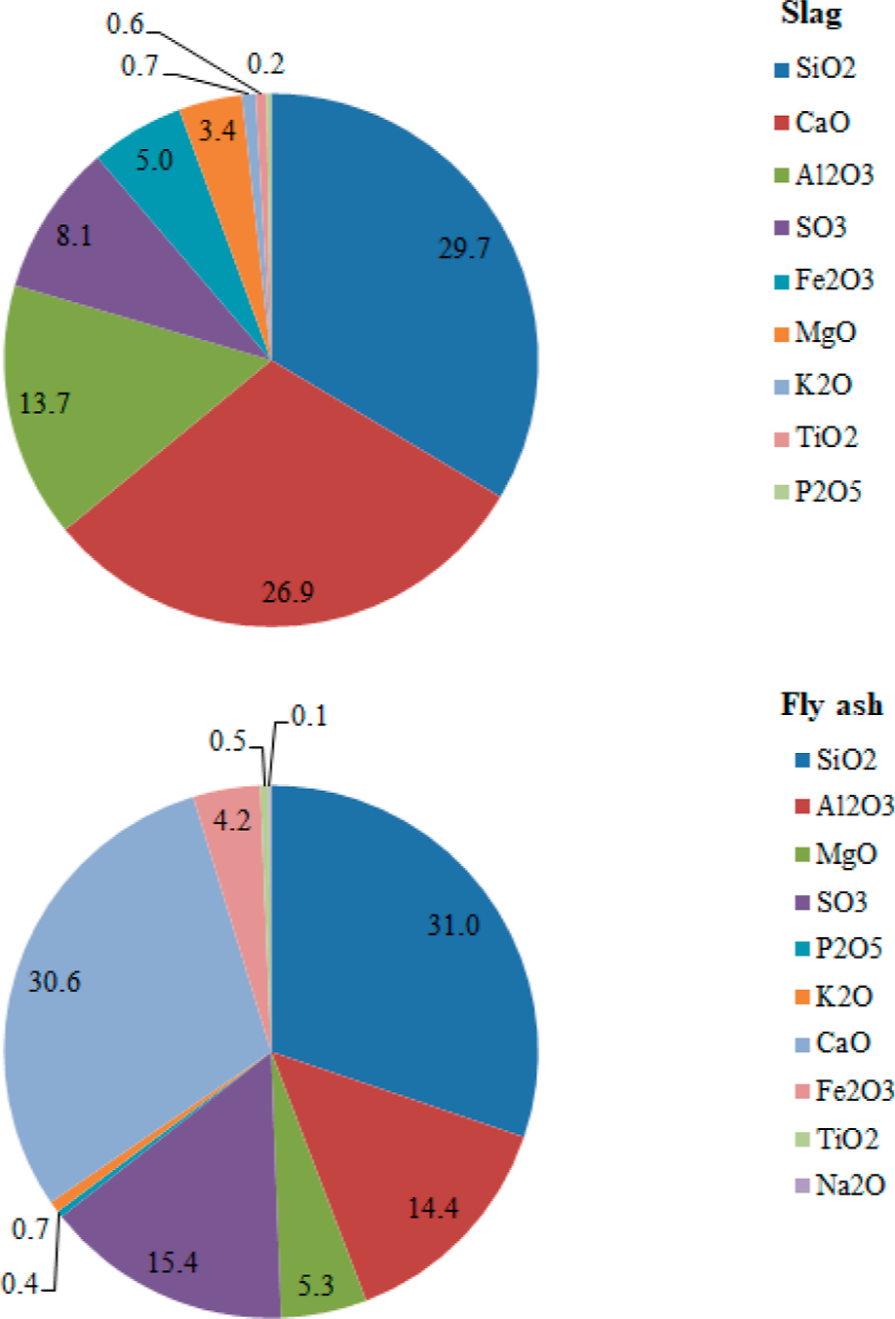
Major and mineral composition of lignite slag and fly ash in percentage.

Distributions of major (>1%) and minor (>0.1%) oxides for LS and FA samples are shown in Figure 2. The oxides analyzed in the LS samples are found to be in the order of SiO_2_ > CaO > Al_2_O_3_ >SO_3_ > Fe_2_O_3_ > MgO > K_2_O > TiO_2_ > P_2_O_5_. The oxides analyzed in the FA samples are found to be in the order of SiO_2_ > CaO > SO_3_ > Al_2_O_3_ > MgO > Fe_2_O_3_ > K_2_O > TiO_2_ > P_2_O_5_ > Na_2_O. Silicon dioxide (SiO_2_) is the most abundant in both LS and FA, with an average value of 29.7 and 31.0% of total mass, respectively. Calcium oxide (CaO) is the second most abundant constituent of both LS and FA samples, with an average value of 26.9 and 30.6%, respectively. The average concentrations of aluminum oxide (Al_2_O_3_) and sulfur trioxide (SO_3_) are found to be higher in FA compared to LS. The average concentrations of iron(III) oxide (Fe_2_O_3_) and titanium dioxide (TiO_2_) are found to be higher in LS compared to FA. The average concentration of potassium oxide (K_2_O) analyzed in LS and FA is found as 0.7%.

### 3.2 Heavy Metal Concentration, ER, and EF

**Figure 3:**
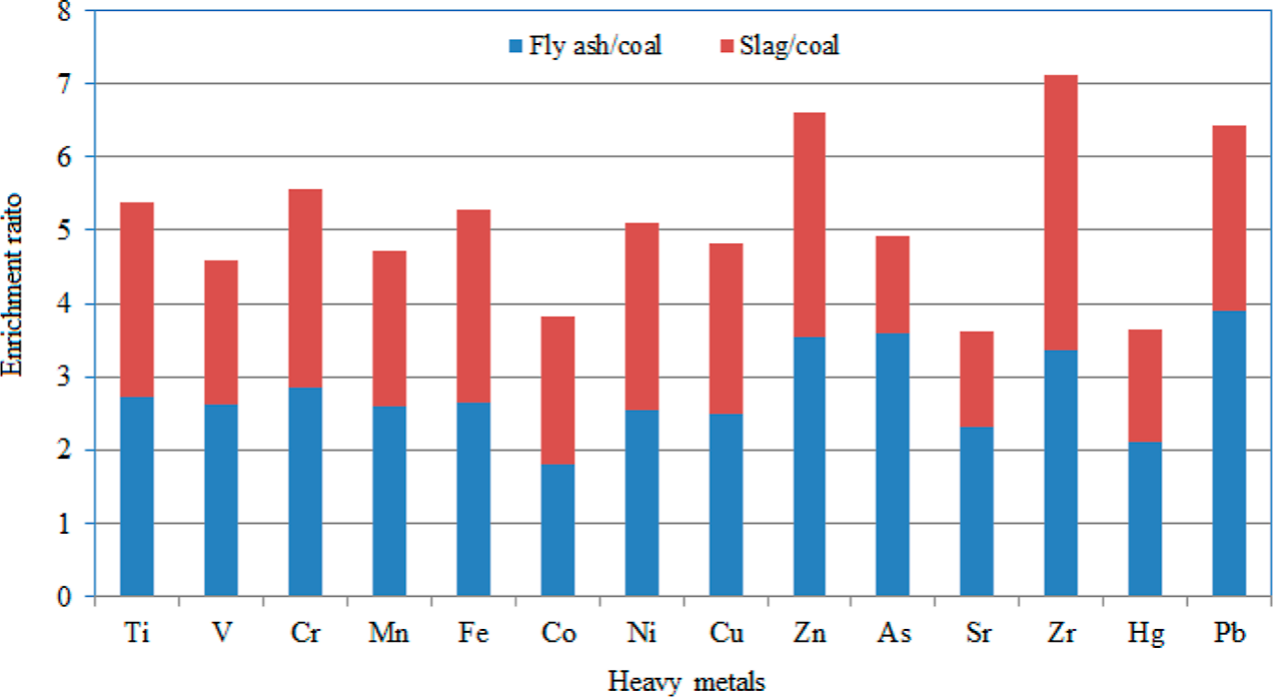
ER for heavy metals in slag and fly ash to lignite coal.

The average concentrations of the heavy metals analyzed in LC, LS, and FA samples are given in Table 1. The relative heavy metal abundances in LC, LS, and FA are in the order of Fe > Ti > Sr > V > Zn > Mn > Cr > Ni > As > Zr > Cu > Co > Pb > Hg; Fe > Ti > Sr > Zn > V > Mn > Cr > Ni > Zr > As > Cu > Pb > Co > Hg and Fe > Ti > Sr > V > Zn > Mn > Cr > Ni > As > Zr > Cu > Pb > Co > Hg, respectively. ER of the heavy metals was calculated as the ratio of the concentration of the heavy metal in LS and FA and to its concentration in LC. The average values of ER for each heavy metal in LS and FA with respect to LC are shown in Figure 3. The average values of the EF estimated for the heavy metals in LC, LS, and FA with respect to Earth’s crustal average are given in Table 2.

**Table 1:** Average Concentration (± Standard Deviation) in Lignite, Slag, and Fly Ash Samples

	elemental concentration (mg/kg)		
	coal	slag	fly ash
Ti	1333.1 ± 136.0	3519.7 ± 265.6	3568.0 ± 416.2
V	145.3 ± 18.6	278.5 ± 29.0	374.7 ± 44.0
Cr	68.9 ± 11.4	181.6 ± 14.6	189.2 ± 23.7
Mn	111.8 ± 24.3	226.0 ± 16.5	272.8 ± 33.0
Fe	13445.0 ± 1415.9	34839.0 ± 1889.0	35211.0 ± 4328.4
Co	13.9 ± 3.7	25.8 ± 2.9	22.5± .8.3
Ni	63.5 ± 11.6	156.7 ± 9.4	155.4 ± 22.4
Cu	18.8 ± 3.2	42.3 ± 2.5	45.3 ± 6.5
Zn	120.2 ± 40.1	328.2 ± 44.7	367.6 ± 95.2
As	39.2 ± 14.4	44.5 ± 4.5	119.2 ± 41.4
Sr	551.5 ± 137.6	698.5 ± 65.6	1227.8 ± 201.3
Zr	32.5 ± 6.0	118.2 ± 8.6	103.8 ± 17.4
Hg	1.3 ± 0.1	1.8 ± 0.4	3.0 ± 0.7
Pb	10.9 ± 3.0	25.9 ± 2.5	40.3 ± 14.7

**Table 2:** EF of Heavy Metals in Lignite, Slag, and Fly Ash to Earth’s Crustal Average

heavy metal	lignite coal	slag	fly ash
Ti	1.6	1.1	1.3
V	8.6	4.5	6.9
Cr	4.4	3.2	3.8
Mn	0.6	0.3	0.5
Fe	1.5	1.1	1.3
Co	4.2	2.1	2.0
Ni	5.7	3.9	4.4
Cu	2.1	1.3	1.6
Zn	7.4	5.7	7.4
As	117.3	37.6	118.6
Sr	9.0	3.0	6.0
Hg	78.3	30.9	59.3
Pb	3.6	2.3	4.3
Zr	1.0	1.0	1.0

Arsenic (As) is markedly toxic and carcinogenic, and it is one of the most important heavy metals that cause both ecological and human health problems.^41^ The average concentration of As analyzed in LC, LS, and FA was found to be 39.2 (17.7–56.8) mg/kg, 44.5 (37.4–50.5) mg/kg, and 119.2 (73.5–217.9) mg/kg, respectively. The average As concentrations are significantly higher than the average concentration of 1.7 mg/kg in Earth’s crust.^42^ The average value of ER (1.3) for As in LS is slightly more than unity, while As in FA is found to be enriched as its ER (3.6) is approximately four times higher than 1. According to values of EF given in Table 1, As has shown extremely high enrichment in LC and FA, while As is found to be very high enriched in slag.

Lead (Pb) is a highly toxic heavy metal that has caused extensive environmental contamination and health problems.^41^ The average concentration of Pb analyzed in LC, LS, and FA was found to be 10.9 (7.8–16.9) mg/kg, 25.9 (23.0–29.1) mg/kg, and 40.3 (23.9–77.4) mg/kg, respectively. The average Pb concentration in LC is lower than the Earth’s crust average of 16 mg/kg,^40^ whereas the Pb concentration in LS and FA is greater than the Earth’s crust average. According to the average values of ER (2.5 for LS and 3.9 for FA), Pb is found to be enriched in both LS and FA as compared to LC. According to values of EF, Pb is found to be moderately enriched in LC, LS, and FA.

Mercury (Hg) is a very toxic heavy metal and extremely bioaccumulative. Its presence adversely affects human health and the environment.^41^ The average concentration of Hg analyzed in LC, LS, and FA was found to be 1.3 (<0.8–1.4) mg/kg, 1.8 (1.2–2.2) mg/kg, and 3.0 (1.7–3.8) mg/kg, respectively. The average Hg concentrations are significantly higher than the Earth’s crust average of 0.083 mg/kg.^42^ According to the average values of ER (1.5 for LS and 2.1 for FA), Hg is enriched in both LS and FA. According to values of EF, Pb is found to be moderately enriched in LC, LS, and FA. According to values of EF, Hg in LC and FA has shown extremely high enrichment, while Hg in LS is found to be very high enriched.

Chromium (Cr, especially Cr^3+^ and Cr^6+^) is a highly toxic and carcinogenic heavy metal for humans, animals, and plants.^41^ The average concentration of Cr analyzed in LC, LS, and FA was found to be 68.9 (52.3–82.2) mg/kg, 181.6 (160.0–199.1) mg/kg, and 189.2 (148.4–214.3) mg/kg, respectively. The average Cr concentration in LC is lower than the Earth’s crust average of 83 mg/kg,^42^ whereas the Cr concentration in LS and FA is approximately two times greater than the Earth’s crust average. According to the average values of ER (2.7 for LS and 2.8 for FA), Cr is found to be enriched in both LS and FA. According to values of EF, Cr is found to be moderately enriched in LC, LS, and FA.

Iron (Fe), which is most important for the growth and survival of almost all living organisms, is the second most abundant heavy metal in the Earth’s crust.^41^ However, a wide variety of harmful free radicals are formed when absorbed iron cannot bind to the protein. This circulating unbound iron can adversely affect human health.^41^ The average concentration of Fe analyzed in LC, LS, and FA was found to be 13,445 (11,530–15,830) mg/kg, 34,839 (31,530–37,400) mg/kg, and 35,211 (27,370–40,600) mg/kg, respectively. The Fe concentrations in LC, LS, and FA are lower than the Earth’s crust average of 46,500 mg/kg.^42^ According to the average values of ER (2.6 for LS and 2.7 for FA), Fe is found to be enriched in both LS and FA. According to values of EF, Fe is found to be slightly enriched in LC, LS, and FA.

Zirconium (Zr) is a naturally abundant heavy metal in the Earth’s crust and is generally considered to have low mobility in soils.^43^ Zr has very low toxicity. The average concentration of Zr analyzed in LC, LS, and FA was found to be 32.5 (24.4–41.8) mg/kg, 118.2 (105.3–129.7) mg/kg, and 103.8 (69.8–125.2) mg/kg, respectively. The Zr concentrations in LC, LS, and FA are lower than the Earth’s crust average of 170 mg/kg.^42^ According to the average values of ER (3.8 for LS and 3.4 for FA), Zr is found to be enriched in both LS and FA.

The accumulation of cobalt (Co) in agricultural areas and water bodies is of concern.^44^ The average concentration of Co analyzed in LC, LS, and FA was found to be 13.9 (<3.0–20.5) mg/kg, 25.8 (22.7–31.5) mg/kg, and 22.5 (12.9–32.9) mg/kg, respectively. The average Co concentration in LC is lower than the Earth’s crust average of 18 mg/kg,^42^ whereas the Co concentration in LS and FA is above the Earth’s crust average. According to the average values of ER (2.0 for LS and 1.8 for FA), Co is found to be enriched in both LS and FA. According to values of EF, Co is found to be moderately enriched in LC, LS, and FA.

Zinc (Zn) is relatively harmless, although in rare cases, Zn toxicity from excessive intake can be harmful to human health.^45^ The average concentration of Zn analyzed in LC, LS, and FA was found to be 120.2 (55.2–156.3) mg/kg, 328.2 (262.8–397.2) mg/kg, and 367.6 (239.8–602.1) mg/kg, respectively. The Zn concentrations in LC, LS, and FA are above the Earth’s crust average of 83 mg/kg.^42^ According to the average values of ER (3.1 for LS and 3.5 for FA), Zn is found to be enriched in both LS and FA. According to values of EF, significant enrichment has been shown by Zn in LC, LS, and FA.

Copper (Cu) toxicity from excessive intake can be harmful to human health. The average concentration of Cu analyzed in LC, LS, and FA was found to be 18.8 (13.6–23.1) mg/kg, 42.3 (39.5–45.6) mg/kg, and 45.3 (32.9–52.5) mg/kg, respectively. The Cu concentrations in LC, LS, and FA are lower than the Earth’s crust average of 47 mg/kg.^42^ According to the average values of ER (2.3 for LS and 2.5 for FA), Cu is found enriched in both LS and FA. According to values of EF, Cu is found to be moderately enriched in LC, while Cu is found to be slightly enriched in LS and FA.

Nickel (Ni) is a potentially toxic heavy metal that can affect multiple organs of living systems.^46^ The average concentration of Ni analyzed in LC, LS, and FA was found to be 63.5 (47.1–79.0) mg/kg, 156.7 (143.2–170.0) mg/kg, and 155.4 (117.3–173.3) mg/kg, respectively. The average Ni concentrations in LC, LS, and FA are higher than the Earth’s crust average of 58 mg/kg.^42^ According to the average values of ER (2.6 for LS and FA), Ni is found to be enriched in both LS and FA. According to values of EF, Ni is found to be significantly enriched in LC, while Ni is found to be moderately enriched in LS and FA.

Manganese (Mn) is the 12th most abundant heavy metal in the Earth’s crust, and excess Mn can cause a wide variety of harmful effects.^47^ Epidemiological data suggest that high Mn concentrations in drinking water may be associated with neurological disorders.^47^ The average concentration of Mn analyzed in LC, LS, and FA was found to be 111.8 (73.3–141.8) mg/kg, 226.0 (207.0–254.9) mg/kg, and 272.8 (214.5–312.8) mg/kg, respectively. The average Mn concentrations in LC, LS, and FA are lower than the Earth’s crust average of 1000 mg/kg.^42^ According to the average values of ER (2.1 for LS and 2.6 for FA), Mn is found to be enriched in both LS and FA. According to values of EF, Mn is found to be slightly enriched in LC, LS, and FA.

Vanadium (V) and strontium (Sr) are not considered serious hazards, but undesired levels of V and Sr can produce harmful effects on health.^48^ The average concentration of V analyzed in LC, LS, and FA was found to be 145.3 (114.4–182.3) mg/kg, 278.5 (222.7–320.7) mg/kg, and 374.7 (299.0–420.4) mg/kg, respectively. The average V concentrations in LC, LS, and FA are higher than the Earth’s crust average of 90 mg/kg.^42^ According to the average values of ER (2.0 for LS and 2.6 for FA), V is found to be enriched in both LS and FA. The average concentration of Sr analyzed in LC, LS, and FA was found to be 551.5 (398.3–790.1) mg/kg, 698.5 (623.8–812.0) mg/kg, and 1227.8 (905.3–1466.0) mg/kg, respectively. The average Sr concentrations in LC, LS, and FA are higher than the Earth’s crust average of 340 mg/kg.^42^ According to the average values of ER (1.3 for LS and 2.3 for FA), V is found to be enriched in both LS and FA. According to values of EF, V and Sr are found to be significantly enriched in LC and FA, while V and Sr are found to be moderately enriched in LS.

Titanium (Ti) is not considered a toxic heavy metal, but it has serious adverse health effects.^49^ The average concentration of Ti analyzed in LC, LS, and FA was found to be 133.1 (1132.0–1522.0) mg/kg, 3519.7 (3164.0–3982.0) mg/kg, and 3568.0 (2739.0–3959.0) mg/kg, respectively. The average Ti concentrations in LC, LS, and FA are lower than Earth’s crust average of 4500 mg/kg.^40^ According to the average values of ER (2.7 for LS and FA), Ti is found to be enriched in both LS and FA. According to values of EF, V is found to be slightly enriched in LC, LS, and FA.

## 4 Conclusions

In this study, heavy metal contents of lignite coal and its wastes, slag and fly ash, were investigated by the EDXRF technique. As a result of the study, it was revealed that the heavy metal concentrations (Ti, V, Cr, Mn, Fe, Co, Ni, Cu, Zn, As, Sr, Zr, Hg, and Pb) in slag and fly ash were enriched according to the concentrations in coal. In addition, the concentrations of Ti, V, Cr, Mn, Fe, Cu, Zn, As, Sr, Hg, and Pb analyzed in fly ash are found to be higher than those analyzed in slag. Also, very toxic heavy metals As and Hg are found to be extremely enriched in coal compared to an average of Earth’s crust. Some heavy metals may leak from ash and slag heaps and contaminate agricultural areas, soil, surface, and groundwater. As a result, the accumulation of these heavy metals in agricultural areas and water bodies is of great concern.

This study emphasizes the transport and accumulation of these heavy metals and raises the need to take effective measures to prevent heavy metals from leaking into the environment such as soil and water bodies.
